# Association of *microRNA-499* rs3746444 Polymorphism with Cancer Risk: Evidence from 7188 Cases and 8548 Controls

**DOI:** 10.1371/journal.pone.0045042

**Published:** 2012-09-10

**Authors:** Fang Wang, Guoping Sun, Yanfeng Zou, Yuanyuan Li, Li Hao, Faming Pan

**Affiliations:** 1 Department of Oncology, The First Affiliated Hospital of Anhui Medical University, Hefei, Anhui, China; 2 Department of Epidemiology and Biostatistics, School of Public Health, Anhui Medical University, Hefei, Anhui, China; Ohio State University Medical Center, United States of America

## Abstract

**Background:**

Owing to inconsistent and inconclusive results, we performed a meta-analysis to derive a more precise estimation of the association between *miR-499* rs3746444 polymorphism and cancer risk.

**Methodology/Principal Findings:**

A systematic search of the Pubmed, Excerpta Medica Database (Embase) and Chinese Biomedical Literature Database (CBM) databases was performed with the last search updated on May 6, 2012. The odds ratio (OR) and its 95% confidence interval (95%CI) were used to assess the strength of the association. A total of 15 independent studies including 7,188 cases and 8,548 controls were used in the meta-analysis. In the present meta-analysis, we found a significant association between *miR-499* rs3746444 polymorphism and cancer risk in the overall analysis (G versus A: OR = 1.10, 95%CI 1.01–1.19, *P* = 0.03; GG+AG versus AA: OR = 1.15, 95%CI 1.02–1.30, *P* = 0.02; GG versus AG+AA: OR = 1.07, 95%CI 0.89–1.28, *P* = 0.50; GG versus AA: OR = 1.13, 95%CI 0.98–1.31, *P* = 0.09; AG versus AA: OR = 1.16, 95%CI 1.02–1.33, *P* = 0.03). In the subgroup analysis by ethnicity, *miR-499* rs3746444 polymorphism was significantly associated with cancer risk in Asian population. In the subgroup analysis by cancer types, *miR-499* rs3746444 polymorphism was significantly associated with breast cancer.

**Conclusions/Significance:**

This meta-analysis suggests a significant association between *miR-499* rs3746444 polymorphism and cancer risk. Large-scale and well-designed case-control studies are necessary to validate the risk identified in the present meta-analysis.

## Introduction

Cancer remains a major cause of mortality worldwide [1]. Based on a new edition of the World Cancer Report from the International Agency for Research on Cancer, about 12.7 million cancer cases and 7.6 million cancer deaths are estimated to have occurred in 2008 [2]. So far, much remains to be learned about the mechanism of carcinogenesis. The increased incidence rate and mortality rate lead researchers to speculate that dietary, infectious, cultural, environmental and/or genetic factors might be implicated in the etiology of the disease. Especially, there is clear evidence that genetic factors play an important role in individual predisposition to cancer [3].

MicroRNAs (miRNAs) are a subset of short, endogenous non-coding RNAs that regulate gene expression at the post-transcriptional level via either translational repression or mRNA degradation [4]. MiRNAs are considered as key regulatory element in gene expression networks, which can influence many biological processes including cell differentiation, proliferation, apoptosis and tumorigenesis [5]. Single nucleotide polymorphism (SNP) is the most common type of genetic variation in human genome. SNPs residing within the miRNA genes could potentially alter various biological processes by influencing the miRNA biogenesis and altering target selection [6]. Furthermore, previous studies have demonstrated that altered expressions of miRNAs play critical roles in cancer development [7]–[8]. Thus, SNPs in miRNAs may in turn influence the individual susceptibility to cancers.

An important polymorphism in the *miR-499* with an A to G change (rs3746444) was identified. The *miR-499* rs3746444 polymorphism involves an A>G nucleotide substitution which leads to a change from A:U pair to G:U mismatch in the stem structure of *miR-499* precursor [9]. To date, a number of case-control studies have been conducted to investigate the association between this polymorphism and cancer risk in diverse populations and multiple types of cancer [9]–[22]. However, these reported results were inconsistent and inconclusive. As far as we know, there is no meta-analysis aimed at investigating the association of *miR-499* rs3746444 polymorphism with cancer risk. Hence, we performed a meta-analysis to derive a more precise estimation of the association to help us better understand the relationship between this polymorphism and cancer risk.

## Materials and Methods

### Identification of eligible studies

To examine the association between *miR-499* rs3746444 polymorphism and cancer risk, a systematic search of the US National Library of Medicine's Pubmed database, Excerpta Medica Database (Embase) and Chinese Biomedical Literature Database (CBM) was performed with the last search updated on May 6, 2012. Keywords used in searches included: “microRNA OR mir OR miRNA”, “cancer OR carcinoma OR tumor OR neoplasm”, “gene OR polymorphism OR allele OR variation”, and “499 OR rs3746444”. Searching was done without restriction on language or publication years.

### Inclusion and exclusion criteria

The inclusion criteria were: 1) evaluation of *miR-499* rs3746444 polymorphism and cancers; 2) a case-control design; 3) sufficient published data for estimating an odds ratio (OR) with 95% confidence interval (CI); 4) only full-text manuscripts were included. Exclusion criteria included: 1) duplication of the previous publications; 2) abstract, comment, review and editorial. When there were multiple publications from the same population, only the largest study was included. When a study reported the results on different ethnicities, we treated them as separate studies. When a study included subjects of different countries, we extracted data separately.

### Data extraction

Information was carefully extracted from all eligible publications independently by two of the authors according to the inclusion criteria listed above. Disagreement was resolved by discussion between the two authors. If these two authors could not reach a consensus, then a third author was consulted to resolve the dispute. Articles identified for this meta-analysis included a case-control study and complete data, including the first author's name, the subjects' region/country, year of publication, cancer types, definition and numbers of cases and controls, allele as well as genotype frequencies in both case and control groups. Their reference lists were searched manually to identify additional eligible studies. If original genotype frequency data were unavailable in relevant articles, a request for additional data was sent to the corresponding author.

### Statistical methods

We used the PRISMA checklist as protocol of the meta-analysis and followed the guideline (Table S1) [23]. Hardy-Weinberg equilibrium (HWE) was evaluated for each study using Chi-square test in control groups. *P*<0.05 was considered representative of departure from HWE. For the meta-analysis, OR and 95% CI were calculated to estimate the association between *miR-499* rs3746444 polymorphism and cancer risk based on reported frequencies of alleles and genotypes in cases and controls. The pooled ORs were performed for allelic comparison (G versus A), dominant model (GG+AG versus AA), recessive model (GG versus AG+AA), homozygote comparison (GG versus AA) and heterozygote comparison (AG versus AA), respectively. The significance of the pooled OR was determined by the *Z*-test. Heterogeneity among studies was assessed by using the Chi-square test based Q-statistic, and, when not statistically significant (based on *P*>0.10), a fixed-effects model (using the Mantel-Haenszel method) was used for the meta-analysis [24]–[25]. Otherwise, the random effect model (using the DerSimonian and Laird method) was used to estimate the summary OR and 95% CI [26]. Heterogeneity was also quantified by using the *I*-squared statistic, *I^2^* = 100%×(Q-df)/Q [27].

### Evaluation of publication bias

Funnel plots were created to graphically display evidence of publication bias, in which the standard error of logarithm for OR was plotted against its OR. An asymmetric plot suggested a possible publication bias. Funnel plot asymmetry was further assessed by the method of Egger's linear regression test [28]. The significance of the intercept was determined by the *t*-test (*P*<0.05 was considered representative of statistically significant publication bias). The intercept *a* provides a measure of asymmetry, and the larger its deviation from zero the more pronounced the asymmetry.

Analyses were performed using the software Review Manager 4.2 (Cochrane Collaboration, http://www.cc-ims.net/RevMan/relnotes.htm/) and Stata version 10 (StataCorp LP, College Station, Texas, USA). A *P* value less than 0.05 was considered statistically significant in the study, and all the *P* values were two sided.

## Results

### Characteristics of studies

**Figure 1:**
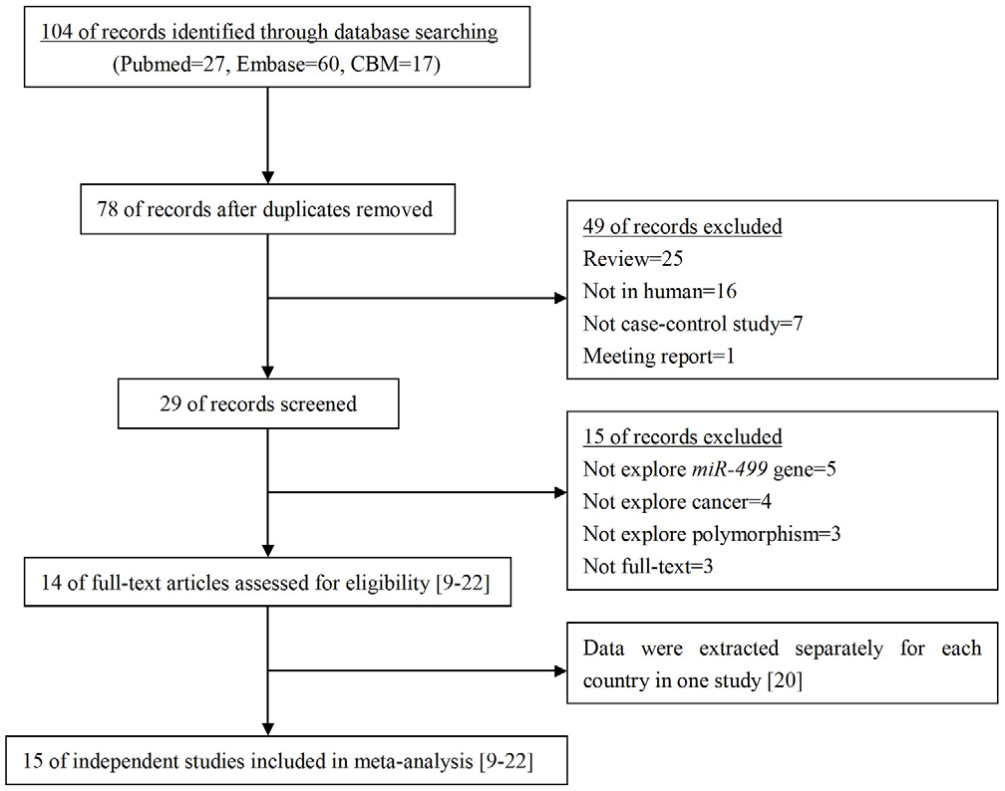
Flow diagram of the study selection process.

There were 104 articles relevant to the searching words (Pubmed:27; Embase:60; CBM:17). The flow chart in Figure 1 summarizes the study selection process. Among these, 14 publications met the inclusion criteria [9]–[22]. In the study of Catucci et al. [20], the ORs were presented separately according to different countries, Germany and Italy. Therefore, we treated them as separate studies. Thus, a total of 15 independent studies including 7,188 cases and 8,548 controls were used in the meta-analysis. Table 1 lists the studies identified and their main characteristics. There were eleven studies of Asian descent [9]–[14], [16], [17], [19], [21]–[22] and four studies of Caucasian descent [15], [18], [20]. The results of Hardy-Weinberg equilibrium test for the distribution of the genotype in control population are shown in Table 1. The genotypes distribution in the controls in 11 of 15 studies was in agreement with HWE [9]–[12], [16], [18]–[22].

**Table 1:** Characteristics of studies included in the meta-analysis.*

ID	Study	Year	Ethnic group	Cancer type	Sample size		*P* for HWE
					Case	Control	
1	Alshatwi et al.[10]	2012	Asian	Breast cancer	100	100	0.227
2	Xiang et al. [9]	2012	Asian	Liver cancer	100	100	0.284
3	Zhou et al. [11]	2012	Asian	Liver cancer	186	483	0.100
4	Min et al. [12]	2011	Asian	Colorectal cancer	446	502	0.453
5	Mittal et al. [13]	2011	Asian	Bladder cancer	212	250	0.020
6	Zhou et al. [14]	2011	Asian	CSCC	226	309	0.005
7	Akkiz et al. [15]	2011	Caucasian	Liver cancer	222	222	0.036
8	George et al. [16]	2011	Asian	Prostate cancer	159	230	0.073
9	Okubo et al. [17]	2010	Asian	Gastric cancer	552	697	0.048
10	Liu et al. [18]	2010	Caucasian	SCCHN	1109	1130	0.441
11	Srivastava et al. [19]	2010	Asian	Gallbaldder cancer	230	230	0.566
12	Catucci et al. [20]	2010	Caucasian (Germany)	Breast cancer	823	925	0.893
13	Catucci et al. [20]	2010	Caucasian (Italy)	Breast cancer	756	1242	0.250
14	Tian et al. [21]	2009	Asian	Lung cancer	1058	1035	0.404
15	Hu et al. [22]	2009	Asian	Breast cancer	1009	1093	0.057

* CSCC, cervical squamous cell carcinoma; SCCHN, squamous cell carcinoma of head and neck; HWE, Hardy-Weinberg equilibrium.

### Main results

The main results of this meta-analysis and the heterogeneity test are shown in Table 2. We first analyzed the association in the overall population. Then in order to obtain the exact consequence of the relationship between *miR-499* rs3746444 polymorphism and cancer susceptibility, stratified analyses by ethnicity and cancer types were performed. When the Q-test of heterogeneity was not significant, we conducted analyses using the fixed effect models. The random effect models were conducted when we detected significant between-study heterogeneity.

**Table 2:** Meta-analysis of *miR-499* rs3746444 polymorphism with cancer susceptibility.*

Comparisons		Sample size		No. of Studies	Test of association				Test of heterogeneity		
		Case	Control		*OR (95%CI)*	*Z*	*P-value*	*Model*	*χ^2^*	*P-value*	*I^2^(%)*
Overall	G vs A	14376	17096	15	1.10(1.01–1.19)	2.17	0.03	R	27.53	0.02	49.1
	GG+AG vs AA	7188	8548	15	1.15(1.02–1.30)	2.34	0.02	R	37.21	0.0007	62.4
	GG vs AG+AA	7188	8548	15	1.07(0.89–1.28)	0.67	0.50	R	22.44	0.07	37.6
	GG vs AA	4929	5966	15	1.13 (0.98–1.31)	1.70	0.09	F	16.64	0.28	15.9
	AG vs AA	6739	8054	15	1.16(1.02–1.33)	2.20	0.03	R	43.45	<0.0001	67.8
Asian	G vs A	8556	10058	11	1.16(1.04–1.28)	2.78	0.005	R	17.17	0.07	41.8
	GG+AG vs AA	4278	5029	11	1.25(1.08–1.45)	3.01	0.003	R	23.36	0.009	57.2
	GG vs AG+AA	4278	5029	11	1.05(0.78–1.41)	0.32	0.75	R	20.89	0.02	52.1
	GG vs AA	2960	3648	11	1.23(1.01–1.50)	2.06	0.04	F	14.28	0.16	30.0
	AG vs AA	4058	4791	11	1.28(1.08–1.52)	2.85	0.004	R	28.71	0.001	65.2
Caucasian	G vs A	5820	7038	4	0.98(0.90–1.07)	0.41	0.68	F	3.58	0.31	16.2
	GG+AG vs AA	2910	3519	4	0.96(0.87–1.06)	0.79	0.43	F	4.79	0.19	37.3
	GG vs AG+AA	2910	3519	4	1.06(0.87–1.29)	0.53	0.60	F	1.49	0.69	0.0
	GG vs AA	1969	2318	4	1.03(0.83–1.28)	0.28	0.78	F	1.03	0.79	0.0
	AG vs AA	2681	3263	4	0.95(0.85–1.06)	0.96	0.34	F	5.77	0.12	48.0
Breast cancer	G vs A	5376	6720	4	1.10(1.01–1.20)	2.09	0.04	F	4.48	0.21	33.0
	GG+AG vs AA	2688	3360	4	1.13(1.01–1.26)	2.19	0.03	F	6.21	0.10	51.7
	GG vs AG+AA	2688	3360	4	1.07(0.71–1.59)	0.31	0.76	R	7.46	0.06	59.8
	GG vs AA	1823	2330	4	1.16 (0.92–1.48)	1.24	0.21	F	4.77	0.19	37.1
	AG vs AA	2552	3196	4	1.16(0.95–1.42)	1.48	0.14	R	7.96	0.05	62.3
Liver cancer	G vs A	1016	1610	3	1.29(0.89–1.87)	1.33	0.18	R	7.12	0.03	71.9
	GG+AG vs AA	508	805	3	1.23(0.94–1.60)	1.54	0.12	F	4.32	0.12	53.7
	GG vs AG+AA	508	805	3	1.34(0.97–1.85)	1.75	0.08	F	4.49	0.11	55.5
	GG vs AA	340	576	3	1.56(0.69–3.48)	1.07	0.28	R	5.97	0.05	66.5
	AG vs AA	390	701	3	1.15(0.86–1.52)	0.95	0.34	F	1.89	0.39	0.0

* OR, odds ratio; vs, versus; R, random effect model; F, fixed effect model.

#### Overall effects for meta-analysis

In the overall analysis, we found a significant association between *miR-499* rs3746444 polymorphism and cancer risk in the allelic contrast, dominant model and heterozygote comparison (G versus A: OR = 1.10, 95%CI 1.01–1.19, *P* = 0.03; GG+AG versus AA: OR = 1.15, 95%CI 1.02–1.30, *P* = 0.02; GG versus AG+AA: OR = 1.07, 95%CI 0.89–1.28, *P* = 0.50; GG versus AA: OR = 1.13, 95%CI 0.98–1.31, *P* = 0.09; AG versus AA: OR = 1.16, 95%CI 1.02–1.33, *P* = 0.03).

#### Subgroup analysis for ethnicity

Subgroup analysis was stratified by ethnicity. The meta-analysis included 11 studies (4,278 cases and 5,029 controls) in Asian population and 4 studies (2,910 cases and 3,519 controls) in Caucasian population.

In Asian population, *miR-499* rs3746444 polymorphism was significantly associated with an increased cancer risk in all genetic models except for recessive model (G versus A: OR = 1.16, 95%CI 1.04–1.28, *P* = 0.005; GG+AG versus AA: OR = 1.25, 95%CI 1.08–1.45, *P* = 0.003; GG versus AG+AA: OR = 1.05, 95%CI 0.78–1.41, *P* = 0.75; GG versus AA: OR = 1.23, 95%CI 1.01–1.50, *P* = 0.04; AG versus AA: OR = 1.28, 95%CI 1.08–1.52, *P* = 0.004). In Caucasian population, no significant association was observed between *miR-499* rs3746444 polymorphism and cancer risk in any genetic model (G versus A: OR = 0.98, 95%CI 0.90–1.07, *P* = 0.68; GG+AG versus AA: OR = 0.96, 95%CI 0.87–1.06, *P* = 0.43; GG versus AG+AA: OR = 1.06, 95%CI 0.87–1.29, *P* = 0.60; GG versus AA: OR = 1.03, 95%CI 0.83–1.28, *P* = 0.78; AG versus AA: OR = 0.95, 95%CI 0.85–1.06, *P* = 0.34).

#### Subgroup analysis for cancer types

Subgroup analysis was also stratified by cancer types. The meta-analysis included 4 studies (2,688 cases and 3,360 controls) based on breast cancer and 3 studies (508 cases and 805 controls) based on liver cancer.

In different types of cancer, *miR-499* rs3746444 polymorphism was significantly associated with an increased risk of breast cancer in the allelic contrast and dominant model (G versus A: OR = 1.10, 95%CI 1.01–1.20, *P* = 0.04; GG+AG versus AA: OR = 1.13, 95%CI 1.01–1.26, *P* = 0.03; GG versus AG+AA: OR = 1.07, 95%CI 0.71–1.59, *P* = 0.76; GG versus AA: OR = 1.16, 95%CI 0.92–1.48, *P* = 0.21; AG versus AA: OR = 1.16, 95%CI 0.95–1.42, *P* = 0.14). No evidence of association was found in any genetic model between *miR-499* rs3746444 polymorphism and the risk of liver cancer (G versus A: OR = 1.29, 95%CI 0.89–1.87, *P* = 0.18; GG+AG versus AA: OR = 1.23, 95%CI 0.94–1.60, *P* = 0.12; GG versus AG+AA: OR = 1.34, 95%CI 0.97–1.85, *P* = 0.08; GG versus AA: OR = 1.56, 95%CI 0.69–3.48, *P* = 0.28; AG versus AA: OR = 1.15, 95%CI 0.86–1.52, *P* = 0.34).

### Evaluation of publication bias

Funnel plot and Egger's test were performed to assess the publication bias of included studies. The results of Egger's linear regression test are shown in Table 3. Egger's test was used to provide statistical evidence of funnel plot symmetry. In the overall analysis, Egger's test detected evidence of publication bias in the allelic contrast (P = 0.022), dominant model (P = 0.006) and heterozygote comparison (P = 0.008). In the subgroup analysis, Egger's test only detected evidence of publication bias in Asian population for dominant model (P = 0.023) and heterozygote comparison (P = 0.019). The shape of the funnel plots revealed similar results.

**Table 3:** Egger's linear regression test to measure the funnel plot asymmetric.*

Groups	Y axis intercept: *a (95%CI)*				
	G vs A	GG+AG vs AA	GG vs AG + AA	GG vs AA	AG vs AA
Overall	2.46 (0.41–4.51)	2.90(1.01–4.79)	−0.63(−2.96–1.70)	0.28(−1.80–2.35)	3.00(0.93–5.07)
Asian	1.79(−1.09–4.67)	2.82(0.50–5.15)	−1.84(−5.72–2.04)	−0.98(−4.20–2.24)	3.17(0.67–5.67)
Caucasian	2.37(−9.21–13.96)	1.00(−9.82–11.83)	4.48(−9.54–18.51)	3.77(−0.15–7.70)	0.42(−11.51–12.35)
Breast cancer	0.92(−9.38–11.22)	2.67(−5.33–10.68)	−1.61(−15.74–12.52)	−0.18(−11.70–11.35)	3.51 (−3.74–10.76)
Liver cancer	6.02(−68.20–80.24)	7.66(−37.26–52.58)	0.89(−33.61–35.40)	1.18(−51.40–53.76)	3.62(−38.43–45.67)

* vs, versus.

## Discussion

In the present meta-analysis with 7,188 cases and 8,548 controls, we found a significant association between *miR-499* rs3746444 polymorphism and cancer risk. In the subgroup analysis of Asian population, *miR-499* rs3746444 polymorphism was significantly associated with an increased cancer risk. Similarly in the subgroup analysis of breast cancer, our data also indicated that this polymorphism might be a risk factor.

In recent few years, several meta-analyses have focused on genetic variants of *miR-146a* and *miR-196a2* genes in the overall cancer risk [29]–[33]. For *miR-146a* rs2910164 polymorphism, Xu et al. [29] and Qiu et al. [31] both showed that no significant associations were found among overall analysis. However, four meta-analyses have all identified that the *miR-196a2* C allele is a low-penetrant risk factor for cancer development, especially with breast cancer and in Asian populations [29], [30], [32], [33]. This finding is similar to that of our meta-analysis, indicating that the two genetic variants (*miR-196a2* rs11614913 and *miR-499* rs3746444) may be functional polymorphisms with potential value in cancer development.

The SNP variation within the miRNA sequence may either weaken or reinforce the binding between miRNA and its target. Therefore, this would probably lead to a corresponding regulation in the target mRNA translation [5], [34]. In a previous study carried out Jazdzewski et al. [35], the data suggested that a common G/C polymorphism within the *pre-miR-146a* sequence decreased the generation of pre- and mature *miR-146a* expression, led to less efficient inhibition of target genes, and contributed to the genetic predisposition to papillary thyroid carcinoma. Furthermore, it has been shown that aberrant expression of miRNA genes could influence the regulation of target genes and involved in tumorigenesis. Recent evidence showed that the cluster of *miR-143* and *miR-145* affected the risk of esophageal squamous cell carcinoma through regulating oncogenic Fascin Homolog 1 (FSCN1) [36]. Alshatwi et al. [10] have explored miRNA expression levels in blood and found that *miR-499* could discriminate breast cancer patients from healthy individuals in postmenopausal patients, which may represent novel biomarker. Based on the above reasons, it can be hypothesized that rs3746444 polymorphism in *miR-499* precursor may alter miRNA processing, and ultimately change the mature miRNA level. Altered miRNA expression may influence cancer susceptibility. As a result, *miR-499* rs3746444 polymorphism could contribute to cancer risk.

In spite of the considerable efforts to explore the possible association between *miR-499* rs3746444 polymorphism and cancer risk, some limitations should be addressed. Firstly, the results should be interpreted with caution as a result of obvious heterogeneity in some comparisons. Secondly, the controls for several studies did not conform to Hardy-Weinberg equilibrium expectations, which may distort the results. However, when these studies that had evidence of departure from HWE were excluded from the analysis, a significant association can still be observed. Thirdly, publication bias existed in some comparisons, which may potentially influence the results of our meta-analysis. Fourthly, lacking sufficient eligible studies limited our further stratified analysis on more types of cancer, such as lung cancer, colorectal cancer and gastric cancer. Fifthly, for each selected case-control study, our results were based on unadjusted estimates, whereas a more precise analysis could be performed if individual data were available.

In conclusion, our meta-analysis suggests a significant association between *miR-499* rs3746444 polymorphism and cancer risk. In the future, large-scale and well-designed case-control studies are necessary to validate the risk identified in the present meta-analysis.

## Supporting Information

Table S1 **Checklist of items to include in this meta-analysis.**
(DOC)
